# Sharp Downregulation of Hub Genes Associated With the Pathogenesis of Breast Cancer From Ductal Carcinoma *In Situ* to Invasive Ductal Carcinoma

**DOI:** 10.3389/fonc.2021.634569

**Published:** 2021-05-21

**Authors:** Yao Wang, Faqing Liang, Yuting Zhou, Juanjuan Qiu, Qing Lv, Zhenggui Du

**Affiliations:** ^1^ Department of Breast Surgery, West China Hospital, Sichuan University, Chengdu, China; ^2^ Laboratory of Public Experimental Platform, West China Hospital, Sichuan University, Chengdu, China; ^3^ Laboratory of Pathology, West China Hospital, Sichuan University, Chengdu, China

**Keywords:** weighted gene correlation network analysis (WGCNA), breast cancer pathogenesis, laser-capture microdissection (LCM), tissue microarrays (TMA), hub gene

## Abstract

**Introduction:**

Breast atypical ductal hyperplasia (ADH) and ductal carcinoma *in situ* (DCIS) are precursor stages of invasive ductal carcinoma (IDC). This study aimed to investigate the pathogenesis of breast cancer by dynamically analyzing expression changes of hub genes from normal mammary epithelium (NME) to simple ductal hyperplasia (SH), ADH, DCIS, and finally to IDC.

**Methods:**

Laser-capture microdissection (LCM) data for NME, SH, ADH, DCIS, and IDC cells were obtained. Weighted gene co-expression network analysis (WGCNA) was performed to dynamically analyze the gene modules and hub genes associated with the pathogenesis of breast cancer. Tissue microarray, immunohistochemical, and western blot analyses were performed to determine the protein expression trends of hub genes.

**Results:**

Two modules showed a trend of increasing expression during the development of breast disease from NME to DCIS, whereas a third module displayed a completely different trend. Interestingly, the three modules displayed inverse trends from DCIS to IDC compared with from NME to DCIS; that is, previously upregulated modules were subsequently downregulated and vice versa. We further analyzed the module that was most closely associated with DCIS (p=7e−07). Kyoto Gene and Genomic Gene Encyclopedia enrichment analysis revealed that the genes in this module were closely related to the cell cycle (p= 4.3e–12). WGCNA revealed eight hub genes in the module, namely, *CDK1, NUSAP1, CEP55, TOP2A, MELK, PBK, RRM2*, and *MAD2L1*. Subsequent analysis of these hub genes revealed that their expression levels were lower in IDC tissues than in DCIS tissues, consistent with the expression trend of the module. The protein expression levels of five of the hub genes gradually increased from NME to DCIS and then decreased in IDC. Survival analysis predicted poor survival among breast cancer patients if these hub genes were not downregulated from DCIS to IDC.

**Conclusions:**

Five hub genes, *RRM2, TOP2A, PBK, MELK*, and *NUSAP1*, which are associated with breast cancer pathogenesis, are gradually upregulated from NME to DCIS and then downregulated in IDC. If these hub genes are not downregulated from DCIS to IDC, patient survival is compromised. However, the underlying mechanisms warrant further elucidation in future studies.

## Highlights

Hub genes, namely *TOP2A, MELK, PBK, NUSAP1*, and *RRM2*, are closely associated with the occurrence and pathogenesis of breast cancer.

The hub genes associated with the progression of breast cancer are gradually overexpressed from normal mammary epithelium to ductal carcinoma *in situ*.

The expression of hub genes tends to decline when breast ductal carcinoma *in situ* further develops to invasive ductal carcinoma.

## Introduction

The main stages of breast disease consist of no atypical cell proliferation, atypical proliferation of ductal or lobular epithelial cells, carcinoma in situ, and invasive cancer. The pathogenesis of breast cancer proceeds from the development of normal mammary epithelium (NME) to simple ductal hyperplasia (SH) and atypical ductal hyperplasia (ADH), subsequently progressing to ductal carcinoma *in situ* (DCIS) and eventually to invasive ductal carcinoma (IDC) (1–4). The prevalence of breast cancer among ADH patients is four-fold that of the general population (5). Moreover, individuals diagnosed with DCIS are at increased risk of IDC (3). If DCIS is not treated, approximately 14–46% of patients develop invasive breast cancer within the next 10 years (1, 6). Although studies have focused on early molecular changes before the onset of aggressive cancer, limited information is available regarding the genetic characteristics of breast cancer precursors.

Breast cancers usually originate from epithelial tissue (7). However, previous tissue assessments have revealed numerous interstitial cells that lead to different outcomes, including fibroblasts, macrophages, and lymphocytes. Cellular heterogeneity is a common challenge in genomic and proteomic tissue analyses; hence, it is necessary to isolate individual cell populations and analyze them separately (8). Currently, the use of laser-assisted microdissection (LCM) of tumor cells facilitates direct microscopic assessment of heterogeneous tissues and yields a rich cell population in the sample. Therefore, LCM represents an extremely sensitive and accurate means of evaluating the pathogenesis of breast cancer in epithelial cells.

Traditional genetic analysis methods include the identification of differentially expressed genes (DEGs); however, this method only compares DEGs between two groups (9). Weighted gene co-expression network analysis (WGCNA) is commonly used to investigate the complex associations between genes and phenotypes (10). WGCNA converts gene expression data into co-expression modules, providing insights into the signaling networks that potentially result in phenotypic traits (11). Most importantly, WGCNA facilitates the analysis of dynamic expression of gene modules or genes associated with disease pathogenesis (12, 13).

In this study, we performed LCM to analyze gene expression in NME cells, SH epithelial cells, ADH epithelial cells, DCIS epithelial cells, and IDC epithelial cells, using WGCNA to further analyze dynamic changes in hub genes during breast cancer pathogenesis. Tissue microarrays (TMAs) consisting of 60 samples derived from different breast diseases were used to evaluate the protein expression levels associated with hub genes. This study aimed to identify dynamic changes in the expression of hub genes associated with breast cancer development, to improve the current understanding of breast cancer pathogenesis.

## Materials and Methods

### Microarray Data

Data from datasets GSE5847, GSE9574, GSE11965, GSE16873, GSE20437, and GSE24506 (n=146 samples) were obtained from the Gene Expression Omnibus (GEO) (www.ncbi.nlm.nih.gov/geo) database for microarray-based gene expression profiling (14). Data for normal terminal ductal lobular units adjacent to breast cancer tissue (n=14) were also included, denoted cancer normal (CN). Microarray data annotation (HG-U133A) was used to match 22283 microarray probes to their corresponding genes. After eliminating probes with more than one target gene and calculating the average expression level of genes corresponding to more than one probe, we finally selected the top 5000 genes with large variance for subsequent analysis.

### Identification of Co-Expression Modules

Clusters with appropriate thresholds were generated using the flashClust tool in the R package (version 3.5) to detect outliers. To establish a predictive gene co-expression network, we set a soft threshold for scale independence of approximately 0.8 to analyze the scale-free topology with inherent module characteristics (15, 16). Modules, identified as sets of genes with high topological overlap, were constructed using the WGCNA algorithm. To ensure high reliability of the results, the minimum number of genes in a module was set to 30.

### Determination of the Association Between Modules and Clinical Traits

By analyzing module–trait associations using the graph function of the R statistical package (version 3.5), modules with common expression patterns were analyzed and interactions of co-expression modules associated with particular traits were identified from correlations between module eigengenes (MEs) and clinical traits. We identified modules most closely associated with the clinical features of breast cancer, including those positively or negatively associated with the clinical characteristics.

### Association Analysis and Hub Genes

WGCNA using the MEs was performed to assess the potential correlation between gene modules and clinical traits (15). MEs were defined as the initial principal components determined *via* principal component analysis, thus summarizing the expression patterns of the module genes into a single characteristic expression profile. Thereafter, the expression of gene modules associated with a type of sample was determined on the basis of gene significance (GS) and module significance (MS) (16). GS for a gene was defined as the −log_10_-transformed p-value of a paired-samples student’s t-test measuring differential gene expression among different types of samples, while MS was indexed as the average GS for all genes in this module.

The eigengene connectivity (KME) was defined as the Pearson correlation coefficient between individual genes and the ME, indicating the distance between the expression profile of a gene and that of the ME. Therefore, the KME quantifies the distance between the gene and the module, determining the module membership of a gene. A pivotal gene is one with high network connectivity in a particular population. In addition, the central genes of each module are highly correlated with the clinical characteristics of each module.

### Method for Determining Hub Genes

Hub genes were determined using scatter plots to further clarify the GS within each module. The connectivity of each gene in each module was calculated, and the gene with the greatest interactions was considered to be the hub gene. Based on the GS and KME, hub genes were selected in accordance with the following criteria: a hub gene had GS>0.2 and KME>0.8.

### Functional Annotation Modules

We carried out database annotations, visualization, and integration to identify sites for enrichment analysis (DAVID, version 6.8, https://david.ncifcrf.gov/home.jsp), including gene ontology (GO) terminology (17) and Kyoto Gene and Genomic Gene Encyclopedia (KEGG) (18) enrichment analysis with respect to molecular function (MF), biological process (BP), and cellular composition (CC).

### Patient Samples

We collected tissues including normal breast, SH, ADH, DCIS, and IDC samples from patients undergoing resection in West China Hospital of Sichuan University. The TMA contained 60 samples from different breast diseases. Normal breast tissue specimens were paired with breast cancer samples. IDC tissue samples were obtained from patients who had been diagnosed for the first time and had not received neoadjuvant chemotherapy. All patients were independently diagnosed by two pathologists. This study was approved by the Ethics Committee of West China Hospital of Sichuan University, and informed consent was obtained from each patient.

### Immunohistochemical (IHC) Staining

TMA sections of 4 μm thickness were placed on a charged slide, deparaffined, and then rehydrated at a reduced alcohol concentration. Antigens were recovered, sealed, and incubated with primary antibodies. After 6 h, glass slides were treated with secondary antibodies and stained using a DAB peroxidase substrate kit (Solarbio, China).

IHC assays were performed using the following antibodies: CDK1, NUSAP1, CEP55, TOP2A, MELK, PBK, RRM2, and MAD2L1 (Affinity Biosciences, dilution 1:200). Allred scores were used to analyze immunohistochemistry. The positive staining intensity (0: negative; 1: weak; 2: moderate; and 3: strong) was multiplied by the staining area (0: <5%; 1: 5–25%; 2: 26–50%; 3: 51–75%; and 4: >75%). The final score (on a scale of 0 to 12) was converted to a scale from 0 to 3 [a score of 0–1 was considered negative (0); 2–4 was considered weakly positive (1); 5–8 was considered medium (2); and 9–12 was considered highly positive (3)]. After immunohistochemical analysis, the sections were scanned to obtain high-resolution (40X) digital images using a 3DHISTECH scanner (Pannoramic, TaiBei) in the pathology laboratory of West China Hospital.

### Western Blot Analysis

Breast tissue extracts were electrophoresed using 10% sodium dodecyl sulfate polyacrylamide gel electrophoresis and then electrotransferred to polyvinylidene fluoride (Solarbio) membranes. The membranes were sealed with 1X phosphate-buffered saline, 0.1% Tween-20, and 5% skim milk (Bio-Rad) and then incubated with primary antibodies at 4°C overnight. Membranes were then washed with 1X TBS-0.1% Tween-20 film and incubated with secondary antibodies at room temperature for 1 h. Autoradiography was performed with a Bio-Rad ChemiDoc XRS+ system.

### Survival Analysis

We analyzed correlations between overall survival (OS) and disease-free survival (DFS) in breast cancer pathogenesis using Kaplan–Meier plots (http://kmplot.com/analysis/) based on upregulation and downregulation of hub genes. This tool provides data on breast cancer, lung cancer, ovarian cancer, stomach cancer, and liver cancer.

### Statistical Analysis

Statistical analyses were performed using the R statistical software version 3.5 (https://www.r-project.org/) with related packages or our customized functions. One-way analysis of variance (ANOVA) was performed to acquire statistical significance (*p<0.05, **p<0.01, ***p<0.001, ****p<0.0001) with GraphPad Prism v8 (GraphPad Software; La Jolla, CA, USA).

Cox proportional hazards analysis was used to detect the relationship between breast cancer mortality and the expression of hub genes in the module; the results were expressed as hazard ratios (HRs) with 95% confidence intervals. The hypothesis of proportional risk was satisfied by the Kaplan–Meier curve test and shown to be statistically significant by log-rank test. SPSS 25.0 (IBM) was used for all statistical analyses.

## Results

### Construction of Gene Co-Expression Modules Associated With Clinical Traits

**Figure 1:**
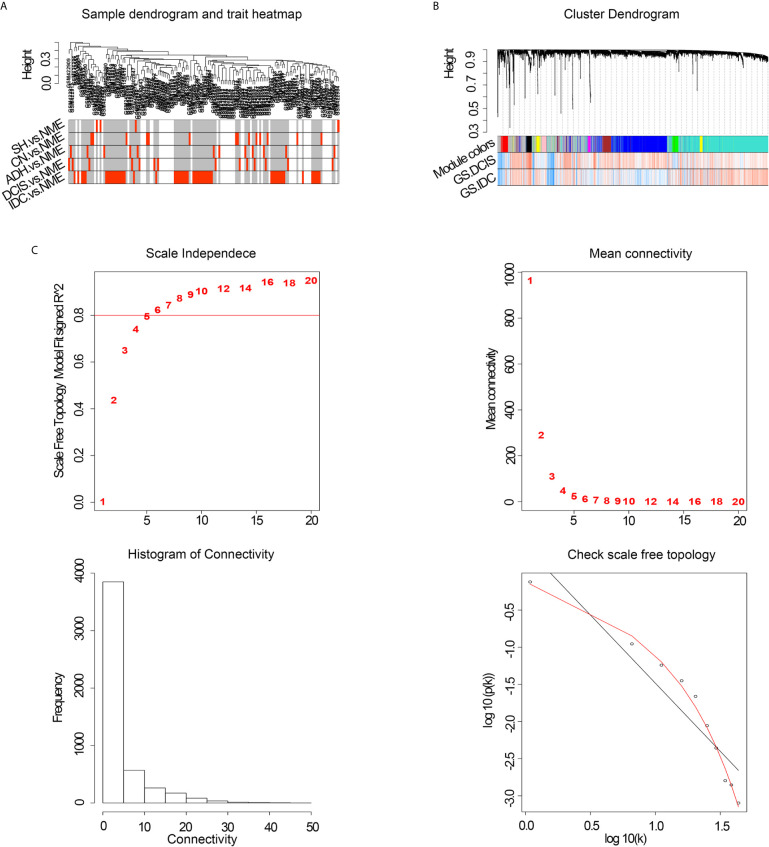
**(A)** Cluster tree of breast cancer samples. **(B)** Analysis of network topology of various soft-thresholding powers. NME, normal mammary epithelium; SH, simple ductal hyperplasia; CN, cancer normal; ADH, atypical ductal hyperplasia; DCIS, ductal carcinoma in situ; IDC, invasive ductal carcinoma; GS, gene significance. **(C)** Hierarchical cluster analysis dendrogram. The gene clustering tree was obtained *via* hierarchical clustering of adjacency-based dissimilarity.

To improve the reliability of the analysis, microarray data for 146 specimens from healthy and diseased individuals, obtained from the GEO database, were pretreated. Co-expression modules were constructed with the most varied 5000 genes in 146 normal breast and pathological breast samples *via* WGCNA. The flashClust tool was used for cluster analysis, and no outlier samples were detected (**Figure 1A**). When the power value was equal to 8, the scale independence approached 0.8 (**Figure 1C**) and predicted a gene co-expression network with scale-free topology and an inherent modular feature. Ten modules were identified from the network of 146 samples *via* hierarchical clustering based on a topological overlap measure dissimilarity measure. A hierarchical clustering system was generated using a color-coded tree diagram for modules, in which DCIS and IDC showed a clear correlation with the black module (**Figure 1B**).

**Figure 2:**
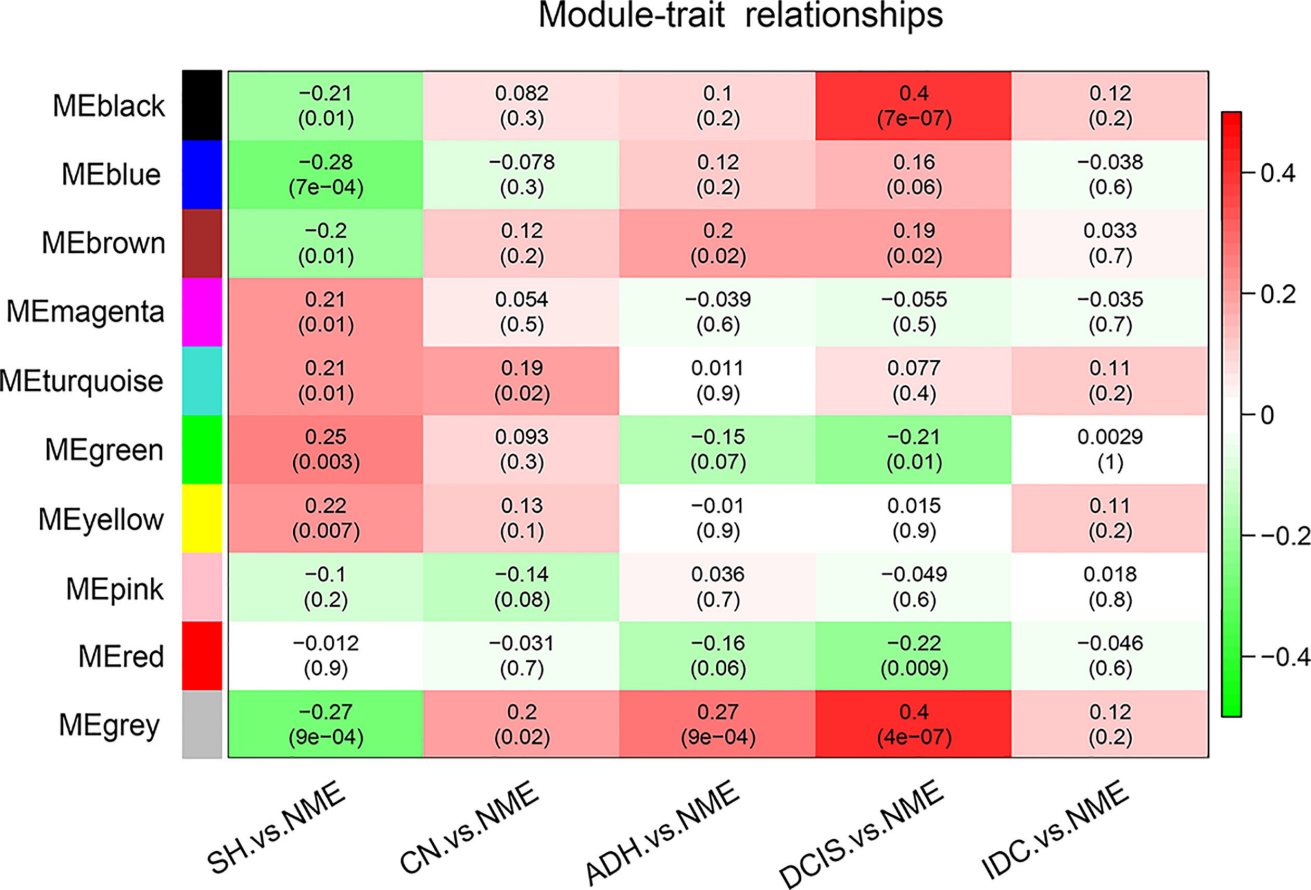
Pearson’s correlation coefficients between MEs and clinicopathological variables. Relationships of the GS measure for weight with breast disease-related genetic and network-based variables of the ten colored modules. NME, normal mammary epithelium; SH, simple ductal hyperplasia; CN, cancer normal; ADH, atypical ductal hyperplasia; DCIS, ductal carcinoma in situ; IDC, invasive ductal carcinoma; ME, module eigengene.

To determine the associations among the ten identified co-expression modules and clinical traits, we determined the Pearson’s correlation coefficient among MEs. Compared with normal tissue, positive correlations with breast cancer pathogenesis for the black (74 genes), blue (845 genes), and brown (181 genes) modules were gradually strengthened as the disease progressed from SH to CN, ADH, and DCIS, with the strongest correlations in DCIS. However, at the stage of invasive cancer, these positive correlations were weaker; for the green (94 genes), magenta (36 genes), and red (92 genes) modules, the positive correlations were gradually weakened in comparison with normal tissue (**Figure 2**). As shown in **Figure 2**, the black module markedly contributed to breast cancer pathogenesis; simultaneously, as shown in the scatter plot (**Supplementary Figure 1**), the genes in the black module were highly correlated with the module of the eigengene, which was highly correlated with the occurrence of DCIS. Thus, the black module was selected for subsequent analysis.

### Functional Enrichment Analysis of Co-Expression Modules

**Figure 3:**
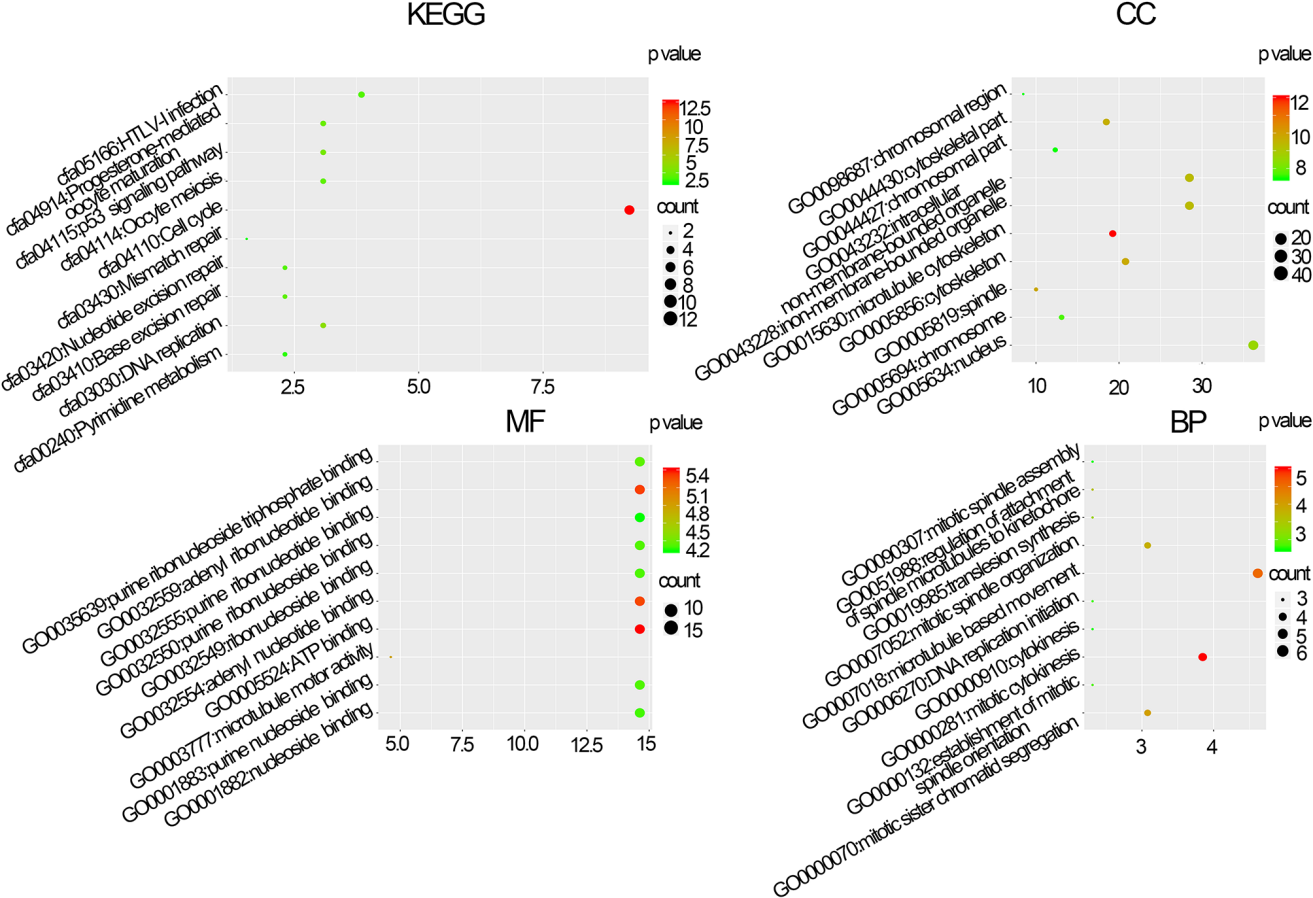
Molecular function (MF), biological processes (BP), cellular composition (CC), and Kyoto Gene and Genomic Gene Encyclopedia (KEGG) pathway enrichment analyses of co-expression modules. The x-axis shows the ratio of genes and the y-axis shows the KEGG pathway terms. The -log10 (p-value) of each term is colored in accordance with the legend.

The BPs in the black module exhibited the highest significant associations with breast cancer pathogenesis and were primarily enriched in the following KEGG pathways: hsa:04110 (cell cycle, p=4.3e–12), hsa:04114 (oocyte meiosis, p=6.25e−04), and hsa:03030 (DNA replication, p=2.92e−04). Regarding MF, genes in the black module were primarily enriched in the following terms: GO:0030554 (adenyl nucleotide binding, p=9.87e–06), GO:0005524 (ATP binding, p=3.75e–06), and GO:0032559 (adenyl ribonucleotide, p=4.58e–06). Regarding CC, the genes in the black module were primarily enriched in the following terms: GO:0015630 (microtubule cytoskeleton, p=2.61e–15), GO:0043228 (non-membrane-bound organelles, p=1.47e−13), and GO:0043232 (intracellular non-membrane-bound organelles, p=1.47e−13). Regarding BP, the genes in the black module were primarily enriched in the following terms: GO:0007049 (cell cycle, p=2.09e–29), GO:0007018 (cell cycle phase, p=4.08e−27), and GO:0022402 (cell cycle process, p=6.77e−26) (**Figure 3**).

### Hub Gene Analysis of Black Module

**Figure 4:**
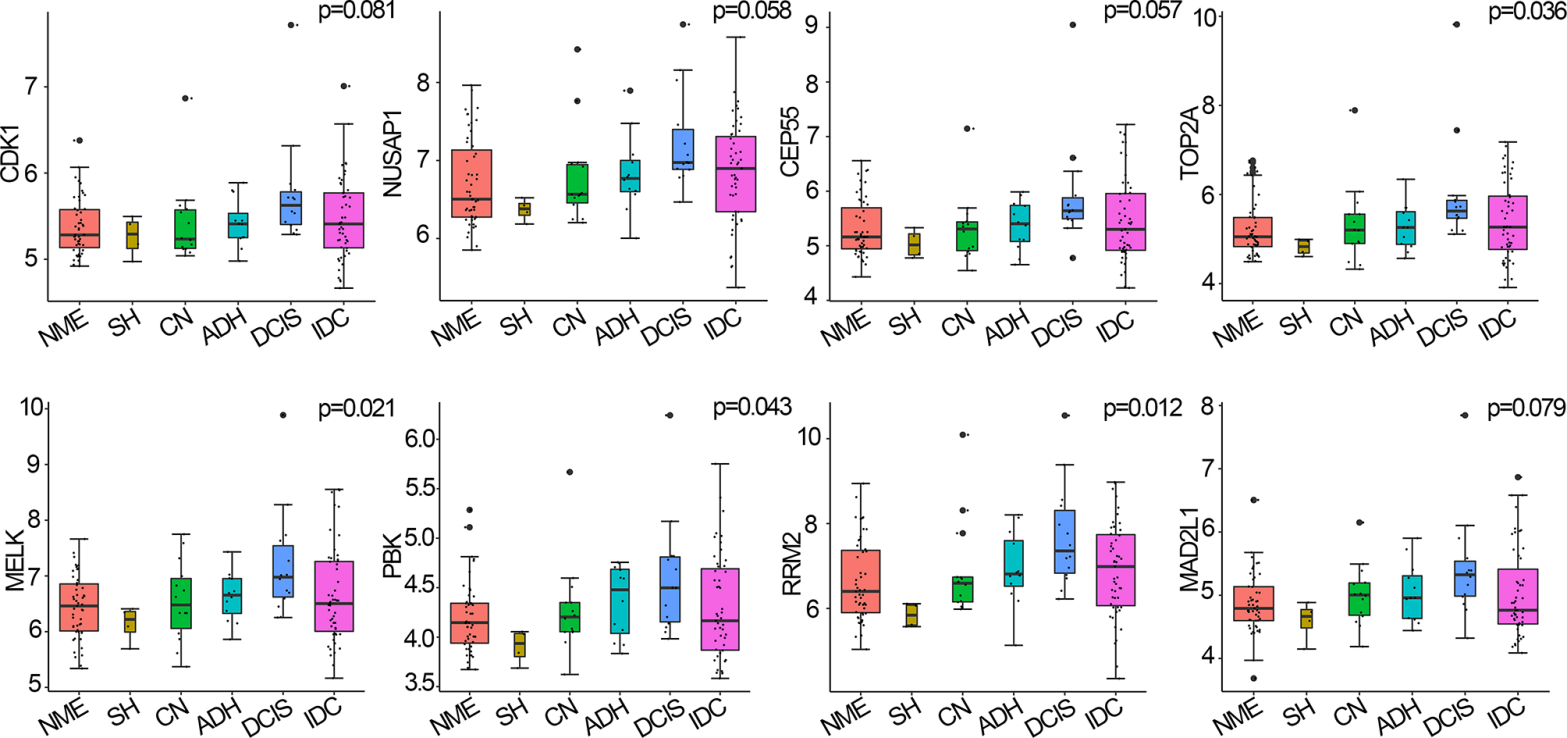
Hub genes expressed in breast cancer and normal tissues, using the Curtis breast dataset (n=146). p-values were calculated *via* log-rank test and p<0.05 was considered significant. NME, normal mammary epithelium; SH, simple ductal hyperplasia; CN, cancer normal; ADH, atypical ductal hyperplasia; DCIS, ductal carcinoma in situ; IDC, invasive ductal carcinoma.

Hub genes are closely related to MEs and are highly associated with breast cancer (here, breast cancer refers to DCIS) (19). Among the 146 samples analyzed herein, *CDK1, NUSAP1*, *CEP55, TOP2A, MELK, PBK, RRM2*, and *MAD2L1* were significantly correlated with the eigengene in the black module and were upregulated in DCIS compared with normal tissues; thus, they were defined as hub genes for breast cancer pathogenesis. Four hub genes, namely *TOP2A* (p=0.036), *MELK* (p=0.021), *PBK* (p=0.043), and *RRM2* (p=0.012), were upregulated during breast cancer progression from normal tissue to DCIS and downregulated during disease progression from DCIS to IDC. *CDK1, NUSAP1, CEP55*, and *MAD2L1* displayed similar, albeit non-significant, trends (p>0.05) to those of the other four hub genes (**Figure 4**).

### Hub Gene Validation by TMA Analysis

**Figure 5:**
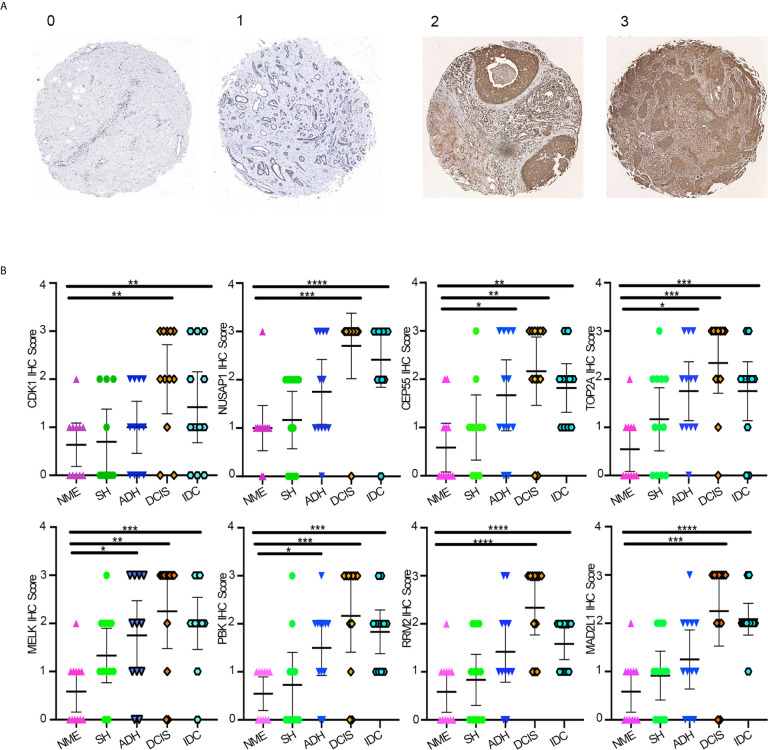
**(A)** Average IHC score scale. **(B)** Average IHC score of eight hub genes using GraphPad Prism v8. One-way ANOVA was performed to determine statistical significance (*p < 0.05, **p < 0.01,***p < 0.001,****p < 0.0001). NME, normal mammary epithelium; SH, simple ductal hyperplasia; ADH, atypical ductal hyperplasia; DCIS, ductal carcinoma in situ; IDC, invasive ductal carcinoma.

Protein expression levels of hub genes were evaluated by IHC staining of eight hub genes on TMAs containing 60 samples derived from different breast disease patients. The clinical characteristics of the patients are summarized in **Table 1**. In the TMA analysis, black module hub genes showed a gradual transition from negative or weakly positive expression to moderate or strong positive staining from NME, SH, and ADH to DCIS; however, in IDC tissues they were negative or weakly positive. The final score was converted to a scale from 0 to 3 (**Figure 5A**). The average IHC scores of the eight hub genes gradually increased from NME to DCIS and then decreased in IDC (**Figure 5B**).

**Table 1:** Clinical characteristics of the patients who provided tissues for the TMA analysis.

	*NME (n=12)*	*SH (n=12)*	*ADH (n=12)*	*DCIS (n=12)*	*IDC (n=12)*	*Total*
*Age (mean ± SD)*	51 ± 3	34 ± 2	38 ± 3	47 ± 4	51 ± 3	60
*Tumor size, cm*	–					
* ≤2*		9	5	4	2	20
* >2*		3	7	8	10	28
*TNM*	–	–	–	–		12
* Stage I*					1	
* Stage II*					3	
* Stage III*					8	
*Nuclear grade*	–	–	–	–		12
* 1*					2	
* 2*					4	
* 3*					4	
* Unknown*					2	
*ER status*	–	–	–	–		12
* postive*					9	
* negative*					3	
*PR status*	–	–	–	–		12
* postive*					9	
* negative*					3	
*Molecular subtype*	–	–	–	–		12
* Luminal A*					3	
* Luminal B*					6	
* HER-2 no-Luminal*					2	
* TNBC*					1	

NME, normal mammary epithelium; SH, simple ductal hyperplasia; ADH, atypical ductal hyperplasia; DCIS, ductal carcinoma in situ; IDC, invasive ductal carcinoma; TNBC, triple-negative breast cancer.

### Hub Gene Validation by Western Blot Analysis

**Figure 6:**
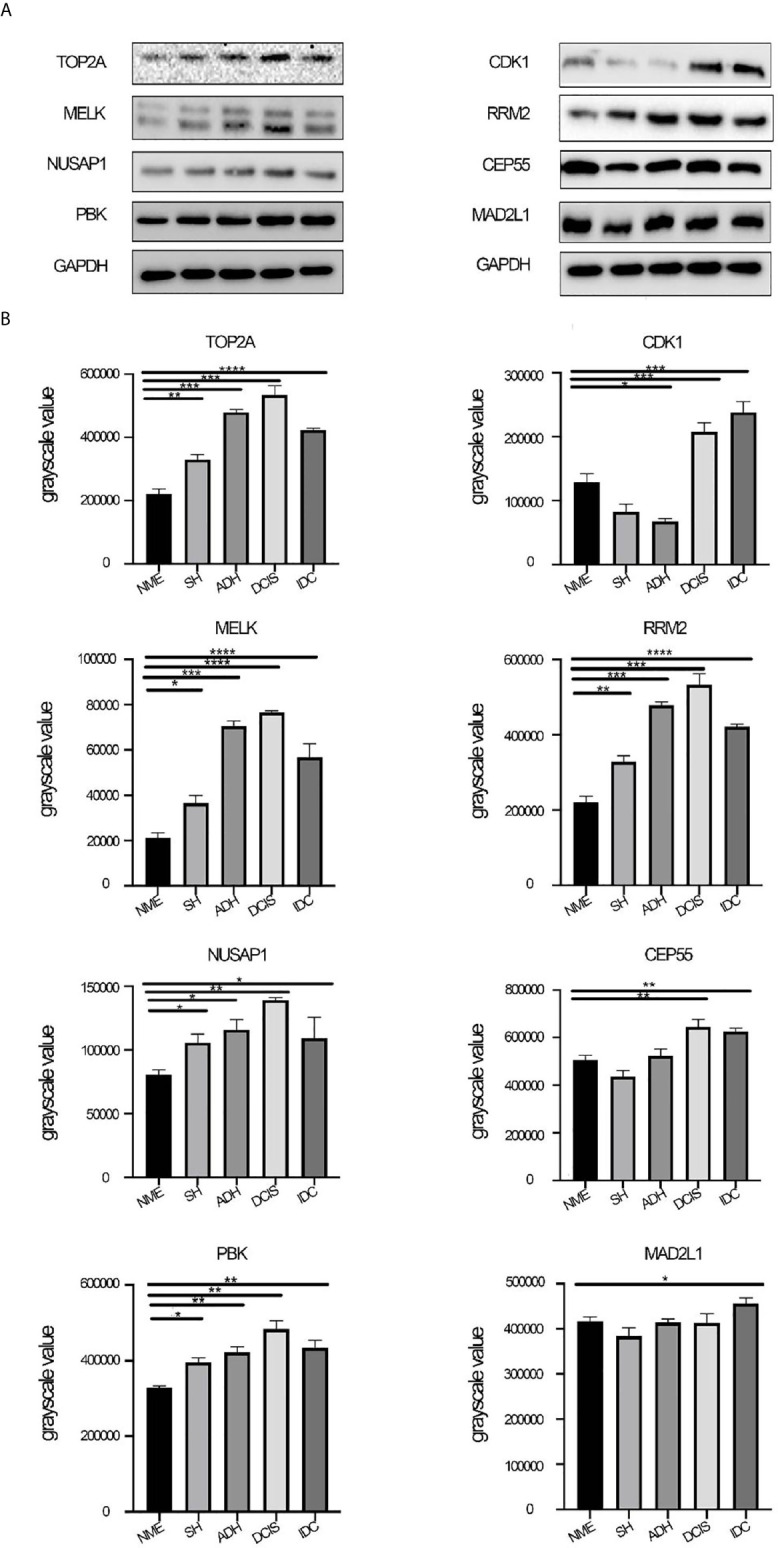
Protein expression levels of hub genes. **(A)** Protein expression levels of CDK1, MELK, CEP55, TOP2A, NUSAP1, PBK, RRM2, and MAD2L1 were determined by western blotting. **(B)** Western blot analysis of CDK1, MELK, CEP55, TOP2A, NUSAP1, PBK, RRM2, and MAD2L1 in tissue from different stages of breast disease, and quantification of the intensity relative to GAPDH. One-way ANOVA was performed to acquire statistical significance (*p < 0.05, **p < 0.01, ***p < 0.001, ****p < 0.0001). NME, normal mammary epithelium; SH, simple ductal hyperplasia; ADH, atypical ductal hyperplasia; DCIS, ductal carcinoma in situ; IDC, invasive ductal carcinoma.

The protein expression of black module hub genes was also validated using western blot analysis. The protein expression levels of hub genes, namely, MELK, TOP2A, PBK, NUSAP1, and RRM2, also gradually increased from NME to DCIS and then decreased in IDC (**Figure 6**). By contrast, the gene expression of CDK1, CEP55, and MAD2L1 showed the opposite trend compared with that of their protein expression. The specific reasons for this have not yet been clarified.

### Survival Analysis

**Figure 7:**
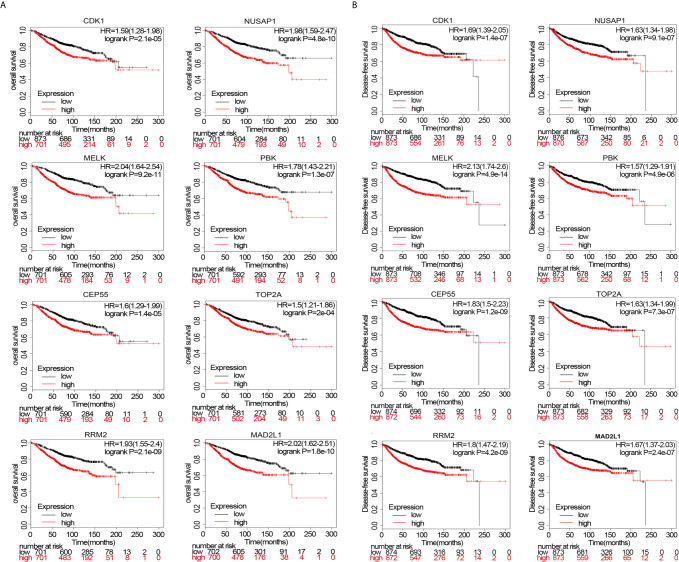
**(A)** overall survival (OS) of patients wherein these genes are expressed. **(B)** disease-free survival (DFS) of patients wherein these genes are expressed.

Survival analysis of upregulated or downregulated hub genes during breast cancer pathogenesis was performed using data from the Kaplan–Meier Plotter website. The HRs for patient OS of hub genes fluctuated between 1.5 and 2.04 (p<0.05). Even *TOP2A*, with the lowest HR (HR=1.5, p=2e−04), could influence OS (**Figure 7A**). Moreover, we simultaneously analyzed DFS and observed the same trend as in the OS analysis. The HRs for DFS of the hub genes fluctuated between 1.57 and 2.13 (p<0.05), indicating that these eight hub genes significantly influenced DFS (**Figure 7B**).

## Discussion

Breast disease pathogenesis is generally a gradual process. In most cases, ADH develops into DCIS, which is a precursor of IDC (3). Particular genes have critical roles in these phenomena. In this study, we identified the key gene module (black module) and confirmed that the expression of hub genes in this module was strongly associated with breast cancer pathogenesis. From normal tissue to DCIS, hub genes in the module were gradually upregulated. However, when the disease progressed from DCIS to IDC, the expression of hub genes displayed a completely opposite trend compared with that observed in the previous stage. This phenomenon has not been reported previously. Many experiments have confirmed that these hub genes are significantly overexpressed in breast cancer tissue compared with normal tissue; however, these studies did not include further dynamic comparisons of hub gene expression in different diseases. Hence, the mechanism underlying the reversal of the trend of expression of these genes remains unclear.

During the progression of breast cancer from SH to DCIS, the black module was closely associated with breast cancer pathogenesis. Functional enrichment analysis revealed that genes in the black module were primarily enriched in cell-cycle-related KEGG pathways, indicating that hub genes in this module are associated with cell proliferation. Healthy cell progression and proliferation proceed through checkpoints during the cell cycle to achieve strict cell cycle regulation; one of the hallmarks of cancer is aberrant cell cycle regulation (20). Furthermore, all cancer cells depend on abnormal metabolism to yield the enormous amounts of energy required to continuously promote cell cycle progression (21, 22). Targeting cell cycle progression to alter metabolism is a crucial therapeutic approach (23), concurrent with our present conclusions. Cell division normally proceeds through the cell cycle, and the ability to sustain aberrant proliferation and dysregulation of the cell cycle is a hallmark of cancer.

Moreover, we assessed breast cancer progression from DCIS to IDC; compared with disease progression in normal breast tissue in DCIS, the black module showed a diametrically opposite trend of a progressive upregulation of gene expression. However, the mechanism underlying this reversal of gene expression during disease progression from DCIS to IDC is unknown. The breast cancer microenvironment includes tumor cells, stromal cells including fibroblasts and vascular and immune cells, and extracellular matrix molecules. When breast cancer progresses to the IDC stage, the microenvironment usually changes (19, 24–26). The interactions between mammary epithelial cells and stromal cells and the changes in their gene expression and enzymatic activity profiles are drivers of disease progression (27–29). Immune cells, including innate immune cells and adaptive immune cells, form an important part of tumor stroma (30). When cancer cells invade the basement membrane, the immune system in the tumor microenvironment (TME) can inhibit tumor growth by destroying or inhibiting the growth of cancer cells (31). When the immune system in the TME cannot eliminate or control the growth of neoplastic cells, cancer progression occurs (32).

The change in trends of hub gene expression may indicate the release of certain substances to the intercellular space, followed by interactions of the TME with tumor cells, leading to changes in expression of hub genes.

We screened hub genes in the black module to elucidate the regulatory mechanism and further assess the evolution of breast cancer. The selected hub genes displayed the same trend as the black module, and if this trend did not decline, it would affect survival. One of the most important genes in breast cancer pathology (C-erbB-2) also displays the same trend (33). The probability of Her-2 overexpression in IDC is approximately 20–30% (34), compared with approximately 50% in DCIS (35). Some studies have reported that this phenomenon results from COX-2 overexpression having a significant association with HER-2 overexpression (14). The COX-2 expression rate is significantly higher in DCIS than in IDC (34); thus, Her-2 overexpression levels are higher in DCIS than in IDC (36). Therefore, it is unclear whether this difference in hub gene expression is regulated by COX-2 or other regulatory mechanisms involving Her-2. This may also be the reason for the change in hub gene expression during disease progression from DCIS to IDC.

Five of the eight hub genes showed the same variation trends with respect to gene and protein expression levels. This suggests that these five genes may have important roles in the initiation of breast cancer. The five genes have also been linked to the development of other cancers. NUSAP1 is overexpressed in hepatocellular carcinoma and glioblastoma (37, 38). Furthermore, NUSAP1 overexpression is associated with deterioration in melanoma and breast and prostate cancers (39, 40). MELK overexpression is associated with tumor aggressiveness and poor outcomes in numerous other cancer types, including glioblastoma (41), astrocytoma (26), and prostate cancer (42). TOP2A upregulation is closely associated with various tumor types, including breast, ovarian, and prostate cancers, because TOP2A catalyzes the cleavage of double-stranded DNA and promotes transcription during mitosis (43). PBK is overexpressed in malignant tumors including Ewing sarcoma, lymphoma, leukemia, melanin tumors, and breast and lung cancers (44–47). RRM2 is overexpressed in cancer and promotes tumor progression (48). Our results show that if these hub genes are still upregulated in the invasive stage of cancer, patient survival is significantly decreased.

In addition, high expression of these five genes was associated with lower survival rates, suggesting that their high expression levels reflect poor prognosis. Hence, it will be of great clinical significance to elucidate the mechanisms underlying the decreased expression of those genes during the transformation from preinvasive to invasive carcinoma, and to further explore the roles of these genes in the initiation and progression of breast cancer. The present results provide a theoretical basis for the prevention and treatment of breast cancer. In further studies, we intend to focus on the mechanisms underlying the downregulation of these genes from DCIS to IDC and identify pathways that could be used to inhibit the high expression of these genes to prevent and treat breast cancer.

## Conclusions

This study shows that hub genes associated with breast cancer pathogenesis, namely *RRM2, TOP2A, PBK, MELK*, and *NUSAP1*, are gradually upregulated from NME to DCIS and then downregulated in IDC. If these hub genes are not downregulated from DCIS to IDC, patient survival is compromised. However, the underlying mechanisms warrant further elucidation in future studies.

## Data Availability Statement

Publicly available datasets were analyzed in this study. This data can be found here: www.ncbi.nlm.nih.gov/geo GSE5847, GSE9574, GSE11965, GSE16873, GSE20437, and GSE24506.

## Author Contributions

YW and FL participated in collecting data, selected papers, carried out the studies, drafted the manuscript, and read, edited, and wrote the final manuscript. ZD designed the project. YW and FL performed IHC and scoring by antibody for TMA slides. JQ, QL, and YZ performed the statistical analyses. ZD read, edited, and approved the final manuscript. All authors contributed to the article and approved the submitted version.

## Funding

This work was supported by projects of the Science and Technology Department, Sichuan Province, China (2018SZ0052 and 2019YFH0146).

## Conflict of Interest

The authors declare that the research was conducted in the absence of any commercial or financial relationships that could be construed as a potential conflict of interest.

## Supplementary Material

Supplementary Figure 1 Scatterplots of gene significance for breast disease *vs.* module membership.

## Abbreviations

ADH, atypical ductal hyperplasia; DCIS, ductal carcinoma in situ; IDC, invasive ductal carcinoma; SH, simple ductal hyperplasia; NME, normal mammary epithelium; LCM, laser-capture microdissection; DEGs, differentially expressed genes; GEO, Gene Expression Omnibus; CN, cancer normal; MEs, module eigengenes; GS, gene significance; MS, module significance; GO, Gene Ontology; KEGG, Kyoto Gene and Genomic Gene Encyclopedia; MF, molecular function; BP, biological processes; CC, cellular composition; OS, overall survival; DFS, disease-free survival; HR, hazard ratio; KME, eigengene connectivity.
