# Supplementary figures and images for: Sharp Downregulation of Hub Genes Associated With the Pathogenesis of Breast Cancer From Ductal Carcinoma In Situ to Invasive Ductal Carcinoma
Source: Front Oncol. 2021 May 21;11:634569. doi: 10.3389/fonc.2021.634569 (PMC8175990; doi:10.3389/fonc.2021.634569)

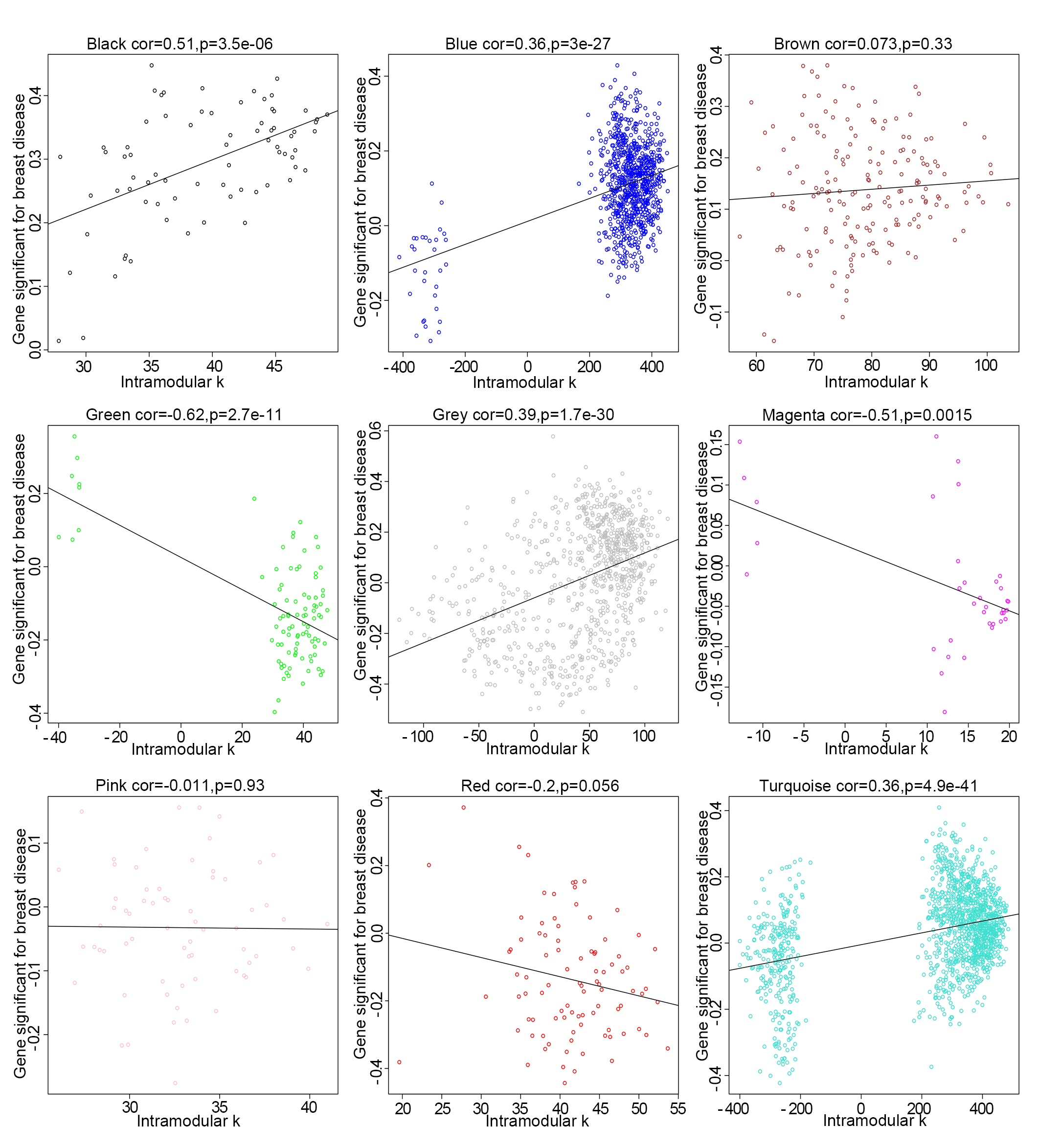

Supplement: Supplementary Figure 1 — Scatterplots of gene significance for breast disease vs. module membership. [file Image_1.tif]
